# The Functional Role of Neural Oscillations in Non-Verbal Emotional Communication

**DOI:** 10.3389/fnhum.2016.00239

**Published:** 2016-05-25

**Authors:** Ashley E. Symons, Wael El-Deredy, Michael Schwartze, Sonja A. Kotz

**Affiliations:** ^1^School of Psychological Sciences, University of ManchesterManchester, UK; ^2^School of Biomedical Engineering, Universidad de ValparaisoValparaiso, Chile; ^3^Department of Neuropsychology, Max Planck Institute for Human Cognitive and Brain SciencesLeipzig, Germany; ^4^Faculty of Psychology and Neuroscience, Department of Neuropsychology and Psychopharmacology, Maastricht UniversityMaastricht, Netherlands

**Keywords:** emotion, nonverbal communication, multisensory, cross-modal prediction, neural oscillations

## Abstract

Effective interpersonal communication depends on the ability to perceive and interpret nonverbal emotional expressions from multiple sensory modalities. Current theoretical models propose that visual and auditory emotion perception involves a network of brain regions including the primary sensory cortices, the superior temporal sulcus (STS), and orbitofrontal cortex (OFC). However, relatively little is known about how the dynamic interplay between these regions gives rise to the perception of emotions. In recent years, there has been increasing recognition of the importance of neural oscillations in mediating neural communication within and between functional neural networks. Here we review studies investigating changes in oscillatory activity during the perception of visual, auditory, and audiovisual emotional expressions, and aim to characterize the functional role of neural oscillations in nonverbal emotion perception. Findings from the reviewed literature suggest that theta band oscillations most consistently differentiate between emotional and neutral expressions. While early theta synchronization appears to reflect the initial encoding of emotionally salient sensory information, later fronto-central theta synchronization may reflect the further integration of sensory information with internal representations. Additionally, gamma synchronization reflects facilitated sensory binding of emotional expressions within regions such as the OFC, STS, and, potentially, the amygdala. However, the evidence is more ambiguous when it comes to the role of oscillations within the alpha and beta frequencies, which vary as a function of modality (or modalities), presence or absence of predictive information, and attentional or task demands. Thus, the synchronization of neural oscillations within specific frequency bands mediates the rapid detection, integration, and evaluation of emotional expressions. Moreover, the functional coupling of oscillatory activity across multiples frequency bands supports a predictive coding model of multisensory emotion perception in which emotional facial and body expressions facilitate the processing of emotional vocalizations.

## Introduction

Effective communication is crucial for the formation and maintenance of social relationships in complex societies. Emotional communication is a complex process where the expression and perception of emotional signals exchanges information about internal affective states. While some of these signals can be expressed through verbal means, much of our emotional communication occurs nonverbally through changes in facial, body, and vocal expressions. Therefore, our ability to perceive and interpret nonverbal expressions of emotion can have a profound impact on the quality of our social interactions, affecting our mental health and wellbeing. To this end, deficits in emotion perception have been observed in a number of neurological and psychiatric conditions (Phillips et al., 2003; Garrido-Vásquez et al., 2011) and may negatively correlate with subjective quality of life in a number of these conditions (i.e., Phillips et al., 2010, 2011; Fulford et al., 2014). Despite this importance, the neural mechanisms and dynamics underpinning the perception of emotional cues within and between sensory modalities is poorly understood. This review explores the functional role of neural oscillations in mediating neural communication within and between sensory modalities in order to facilitate the detection, integration, and evaluation of emotional expressions.

Emotions are commonly defined as brief, coordinated neural, physiological, and behavioral responses to relevant events (Scherer, 2000). These responses can manifest behaviorally as changes in facial expression, body language, tone of voice (prosody), or any combination thereof. Thus, emotion perception can be described as the process of detecting salient signals, integrating those signals with prior knowledge of emotional meaning, and evaluating the integrated representation within the context of the current environment. According to current models, emotion perception of visual (Adolphs, 2002a; De Gelder, 2006), auditory (Schirmer and Kotz, 2006; Wildgruber et al., 2009; Kotz and Paulmann, 2011), and audiovisual (Brück et al., 2011) signals unfolds in three fast yet distinct stages: detection, integration, and evaluation.

The first stage consists of early perceptual processing in what are traditionally considered modality-specific cortices. For visual expressions of emotion, this includes regions of the occipito-temporal cortex, most notably the fusiform gyrus (Adolphs, 2002a,b; Vuilleumier and Pourtois, 2007) with distinct subregions of the fusiform gyrus responding preferentially to facial and body expressions (Schwarzlose et al., 2005), and extriate body area (Grèzes et al., 2007; Kret et al., 2011; Meeren et al., 2013). Early detection of complex acoustic cues occurs in the belt region of the primary auditory cortex (Woods and Alain, 2009) and later in multiple voice-sensitive areas in the temporal lobe (Belin et al., 2004; Wiethoff et al., 2008; Ethofer et al., 2009, 2011; Pernet et al., 2015).

Following the extraction of low-level visual and acoustic features, a more detailed representation of the emotional expression is generated in the superior temporal sulcus (STS). Evidence from neuroimaging research suggests a functional subdivision within the STS with face-sensitive regions in the posterior terminal ascending branch and voice-sensitive regions in the trunk section (Kreifelts et al., 2009). Functional differentiation between middle and anterior regions of the STS has also been noted during the perception of emotional vocal expressions (Kotz and Paulmann, 2011). Receiving input from both visual and auditory cortices, the STS also plays a key role in audiovisual integration (i.e., Calvert et al., 2000, 2001; Beauchamp et al., 2004a,b; Stevenson et al., 2007; Stevenson and James, 2009). To this end, facial and vocal expressions activate overlapping face- and voice-sensitive regions within the STS, suggesting that the STS is essential for the integration of audiovisual emotional information (Robins et al., 2009; Watson et al., 2013, 2014).

In the final stage, the behavioral and motivational significance of the expression is interpreted and evaluated within the inferior frontal gyrus (IFG; Frühholz and Grandjean, 2013a) and orbitofrontal cortex (OFC; Adolphs, 2002b; Kotz et al., 2012). Involved in the processing of reward and punishment (Kringelbach and Rolls, 2004; Rolls, 2004), the OFC is thought to be involved in the representation of stimulus value across sensory modalities. Thus, during emotion perception, the OFC may be responsible for evaluating the emotional value of the expression within the context of the current environment.

In addition to these cortical regions, many studies also support a key role for subcortical structures such as the amygdala and basal ganglia in the perception of emotions. For example, the amygdala has been implicated in the processing of facial (i.e., Phillips et al., 1997; Blair et al., 1999; Whalen et al., 2001, 2013; Williams et al., 2004), body (Hadjikhani and de Gelder, 2003; Grèzes et al., 2007), and vocal (Fecteau et al., 2007; Frühholz and Grandjean, 2013b) expressions in both early and late stages of emotion perception. Studies also support a key role for the basal ganglia in the processing of facial (Adolphs, 2002b) and vocal (Kotz et al., 2003) expressions. Furthermore, deep brain stimulation of the basal ganglia, specifically the subthalamic nucleus, can impair emotion perception from facial and vocal expressions (Péron et al., 2010a,b). Given the importance of the basal ganglia in other aspects of emotion processing (i.e., subjective feeling and production of emotional expressions), it has been proposed that the basal ganglia coordinates the synchronization of different components of emotion processing (Péron et al., 2013). While the amygdala appears to be involved in both the early and late stages of emotion perception, consistent with dual-pathway models of emotion processing (LeDoux, 1996), basal ganglia activity is more often observed in the later stages as a function of attention (Kotz et al., 2012).

In sum, the perception of emotion from facial, body and vocal expressions involves a distributed neural network of cortical and subcortical structures. The question then becomes, how does the brain selectively attend to and integrate these signals across space and time in order to give rise to a unified representation of an emotional expression?

The investigation of such rapid online processing of dynamic changes in sensory input requires adequate methods to capture neural information processing in real time. Electroencephalography (EEG) and magnetoencephalography (MEG) are particularly well suited for the study of emotion perception due to their millisecond temporal resolution.

Results from event-related potential (ERPs) suggest differentiation between emotional and neutral facial expressions within 120 ms of stimulus onset (Eimer and Holmes, 2002; Eimer et al., 2003). The time course of emotion-related effects on evoked responses to vocal expressions depends on the stimulus type with earlier effects for affective bursts such as laughs and screams (Liu et al., 2012) compared to changes in emotion prosody (Paulmann and Kotz, 2008; Paulmann et al., 2012, 2013; Pell et al., 2015). These early ERP effects are thought to reflect rapid detection of salience. Visual (Stefanics et al., 2012) and auditory (Schirmer et al., 2005) deviance detection, in the form of the mismatch negativity (MMN) is observed at approximately 200 ms, supporting the idea that integration of emotional signals occurs at this stage. Across domains, emotional expressions also elicit a sustained positivity (the late positive component or LPC) beginning between 300–400 ms post-stimulus, reflecting the interpretation and evaluation of emotional significance (visual, Eimer and Holmes, 2007; auditory, Paulmann et al., 2013).

Taken together, findings from ERP studies largely support the staged models of visual and auditory emotion perception and further establish a time course for the stages of detection, integration, and evaluation of emotional expressions. While studies of ERPs have undoubtedly advanced our understanding of the time course and neural bases of emotion perception, they can only provide limited insight into the dynamic interaction within and between nodes of functional neural networks. That is, we know substantially more about when and where certain processes may occur, than about *how* these processes arise and unfold within the human brain.

Neural oscillations, which reflect rhythmic fluctuations in the synchronization of neuronal populations, provide a measure of the dynamic interactions within and between regions involved in the different stages of emotion perception. Changes in oscillatory activity are commonly analyzed by treating the on-going EEG (or MEG) signal as the sum of pure sinusoids, which are separated into characteristic frequency bands each associated with distinct cognitive and computational operations. Decomposing the EEG/MEG signal into its constituent sinusoids, allows for the measurement of changes in power (amplitude) and phase within and between each frequency band, at different time points and in different brain regions. While increases or decreases in power—referred to as event-related synchronization (ERS) or desynchronization (ERD) respectively—indicate changes in neural synchronization within a specific node or region, phase coherence across brain regions reflects synchrony between brain regions that make up a functional neural network (Bastiaansen et al., 2012). According to one hypothesis, phase coherence enables the effective communication between neuronal populations (Fries, 2005). Moreover, cross-frequency coupling may facilitate the integration of information across different spatial and temporal scales (Canolty and Knight, 2010). Thus, neural oscillations can provide an index of the dynamic interaction between brain regions involved in emotion perception as well as a plausible mechanism by which the brain can integrate rapidly changing emotional information from facial, body, and vocal expressions. To date, the majority of studies investigating emotion perception have focused solely on power changes. Thus, this review will primarily focus on ERS and ERD, while noting the critical importance of phase coherence and cross-frequency coupling in elucidating the functional dynamics of emotion perception within and between sensory modalities.

## Perception of Emotion from Facial Expressions

Facial expressions are by far the most commonly studied means of emotional communication. In a typical study, participants are presented with images of facial expressions and asked to respond to the emotion (explicit) or identity/gender (implicit) of the face. Using this type of paradigm, studies have found changes in oscillatory activity across multiple frequency bands during the perception of emotion from facial expressions.

### Delta

Delta oscillations have been implicated in a wide range of processes including the perception of faces and facial expressions (Knyazev, 2012; Güntekin and Başar, 2015). While frontal delta synchronization is characteristic of many more “cognitive” tasks, face processing is associated with delta synchronization over more posterior regions (Güntekin and Başar, 2009). Moreover, emotional expressions appear to induce stronger delta synchronization than neutral expressions over occipito-parietal regions, which is suggested to reflect stimulus updating (Balconi and Lucchiari, 2006; Balconi and Pozzoli, 2007, 2008, 2009). Effects of emotion on delta oscillations have also been observed over fronto-central regions, correlating with behavioral measures emotional involvement (Knyazev et al., 2009b). Of note here is that the studies observing occipito-parietal delta synchronization have typically used passive viewing paradigms while Knyazev et al. (2009a) used both implicit (gender identification) and explicit (emotion categorization) tasks. Thus, emotion may differentially affect delta responses to facial expressions depending on task demands. Together these findings suggest a role for delta oscillations in the perception of emotional facial expressions; yet the functional significance of delta synchronization in this context remains unclear. Further research is needed in order to determine more precisely the functional role of delta oscillations within the context of emotion perception.

### Theta (4–7 Hz)

Most commonly associated with memory encoding and retrieval (Klimesch, 1999), theta band oscillations are thought to play a key role in the processing of emotion (Knyazev, 2007). To this end, recent studies have shown enhanced theta synchronization for emotional compared to neutral facial expressions, suggesting that theta oscillations may facilitate the rapid encoding of emotionally salient sensory information. For instance, Balconi and colleagues have observed enhanced theta synchronization over predominantly right frontal regions of the scalp between 150–250 ms extending into the later time window of 250–350 ms which they suggest reflects the orienting of attention toward the emotional significance of the stimulus during the early stages of conceptual processing (Balconi and Lucchiari, 2006; Balconi and Pozzoli, 2009). Similar results were reported by Knyazev et al. (2009b, 2010), who found increased early theta ERS over right frontal regions during the implicit processing of emotional facial expressions, that is, when participants performed a gender categorization task in which attention was directed away from the emotional content of the stimulus. Furthermore, these authors observed a second distinct theta ERS between 230–350 ms that was greater when the emotional content of the stimulus was processed explicitly during an emotion categorization task. Source localization revealed differential activation in the right parietal cortex (angry) and insula (happy) in the early time window and left temporal lobe (angry) and bilateral PFC (happy) in the later time window. Interestingly, some studies have also observed theta synchronization over more posterior (occipital and occipito-parietal) regions within a similar (early) time window (i.e., Başar et al., 2006; Balconi and Pozzoli, 2007, 2008), an effect that increases as a function of visual awareness (Zhang et al., 2012). However, the extent to which this theta synchronization is emotion-specific may be called into question on the basis of a study by González-Roldan et al. (2011) showing no effect of emotion on theta synchronization during an explicit task. Instead, the authors observed a main effect of intensity on theta ERS between 200–400 ms over frontal, central, and parietal regions. This may suggest that theta synchronization in response to emotional facial expressions may reflect facilitated encoding of the biological or motivational significance rather than the emotional quality of the expression *per se*. That is, emotional expressions (relative to neutral expressions) contain more behaviorally relevant sensory information, which reduces uncertainty, resulting in stronger neural synchronization in the theta frequency. This enhanced theta synchronization facilitates the dynamic between brain regions involved in the early detection and integration of static emotional facial expressions.

### Alpha (8–12 Hz)

As first noted by Berger (1929), neural oscillations in the alpha frequency band show strong synchronization over occipital regions in the absence of visual stimulation (i.e., with eyes closed). Based on further evidence showing alpha ERS over cortical regions not necessary for a given task, alpha synchronization was initially taken as an indicator of cortical idling (Pfurtscheller et al., 1996). However, more recent hypotheses suggest that alpha synchronization serves an active role in the inhibition of task-irrelevant brain regions (Klimesch et al., 2007; Jensen and Mazaheri, 2010). The rhythmic fluctuation of alpha oscillations thus produces temporal windows in which neurons are more or less likely to fire. Larger amplitudes (reflecting stronger inhibition) result in smaller temporal windows and thus more precise timing of neuronal firing. Smaller amplitudes, associated with release of inhibition, result in greater cortical excitability over longer temporal intervals. Within the context of emotion perception, alpha oscillations may be involved in the selective attention to emotionally salient social cues through active inhibition of task-irrelevant regions and pathways. It is notable, however, that many studies using static faces have found no difference in alpha synchronization between emotional and neutral expressions (Balconi and Lucchiari, 2006; Balconi and Pozzoli, 2007, 2008, 2009). Differences in alpha power emerge more reliably when comparing expressions of positive and negative valence. While perception of negative emotional expressions was associated with right-lateralized alpha ERD, perception of positive emotional expressions was associated with left-lateralized alpha ERD (Balconi and Ferrari, 2012). Although greater when facial expressions were presented supraliminally, these valence-specific differences were also observed when expressions were presented subliminally. Further support for these findings comes from a study by Del Zotto et al. (2013) showing valence-specific lateralization of frontal alpha power in a patient with cortical blindness. Results from this study showed that alpha ERD was greatest for fear compared to happy expressions over right frontal regions even though the patient could not report seeing the stimuli. Other evidence suggests that alpha synchronization over posterior regions may also differentiate between stimuli of negative and positive valence (Başar et al., 2006), though this effect was only observed when selecting the stimuli with the most extreme valence ratings for analysis.

While studies using static stimuli highlight the roles of valence in alpha responses to facial expressions, they have two important limitations. Firstly, in naturalistic human communication, facial expressions are inherently dynamic and therefore the extent to which these findings would be valid in naturalistic settings is unclear. Secondly, although providing a rough estimate as to the topographical distribution of alpha ERD, these studies only provide limited insight into the patterns of functional connectivity underpinning the perception of emotion from facial expressions. Addressing these issues, a recent MEG study used dynamic facial expressions to explore changes in spatial connectivity during emotion perception (Popov et al., 2013). Findings from this study provide evidence for two stages of upper alpha desynchronization during facial emotion perception: a pre-recognition stage associated with increased alpha power over frontal and sensorimotor regions and decreased alpha power over occipital regions followed by a post-recognition stage associated with the reversed pattern. Moreover, these power changes were associated with inverse patterns of functional connectivity, suggesting that alpha synchronization and desynchronization may regulate the exchange of information between visual and sensorimotor. That these effects were stronger in response to emotional compared to neutral expressions implies that emotion may enhance the functional coupling, facilitating recognition of facial expressions of emotion.

### Beta (13–30 Hz)

Oscillatory activity in the beta frequency is typically associated with sensorimotor processing (Brovelli et al., 2004). However, recent evidence suggests a broader role for beta synchronization in the maintenance of current sensory, motor, and cognitive sets (Engel and Fries, 2010). Beta band oscillations have also been implicated in the perception of emotion from facial expressions. However, the direction, time course, and topography of beta modulation vary considerably between studies. For example, Güntekin and Başar (2007a) found increased beta power for angry compared to happy expressions over frontal and central regions. In a similar study including occipital electrodes, however, the authors found no main effect of emotion on beta band activity (Güntekin and Başar, 2007b). Thus, it seems that only fronto-central beta synchronization reflects differentiation between emotional and neutral facial expressions. Other evidence suggests that such differences in beta synchronization may also be modulated by attention. To this end, asymmetry in resting-state parietal beta band activity has been negatively correlated with attentional bias towards angry facial expressions (Schutter et al., 2001).

Given the importance of beta oscillations in the perception of biological motion, which is thought to be critical for social cognition in naturalistic environments (Pavlova, 2012). Jabbi et al. (2015) used MEG to compare evoked beta band activity in response to dynamic and static facial expressions. Perhaps unsurprisingly, greater beta power was observed for dynamic compared to static facial expressions in occipital, superior temporal and sensorimotor cortices. When comparing dynamic emotional to neutral expressions, the authors found stronger beta power in regions such as the amygdala, STS, and OFC. Furthermore, beta power in the left STS was negatively correlated with the time course of fearful facial expressions but positively correlated with the time course of happy facial expressions. These emotion-specific differences suggest that the observed changes in beta power were not solely due to the processing of biological motion. Although this study investigates evoked rather than induced oscillatory activity, its findings support a putative role for beta oscillations—particularly within the STS—in tracking the temporal dynamics of facial expressions of emotion.

### Gamma (>30 Hz)

Reflecting neuronal communication on a more local scale, gamma oscillations have been implicated in a number of cognitive processes including feature integration (Singer and Gray, 1995) and sensory selection (Fries et al., 2002). Within the context of emotion perception, event-related gamma synchronization has been commonly used to explore the functional dynamics underpinning the conscious and unconscious processing of emotional facial expressions. Studies investigating the spatial and temporal dynamics of emotion perception support a dual-pathway model of emotion perception consisting of a cortical and subcortical pathway (i.e., LeDoux, 1996). Accordingly, in an MEG study, Luo et al. (2007, 2009) have reported that fearful expressions elicit early gamma band activity in the amygdala followed by later responses in the occipital, parietal, and prefrontal cortices. These authors have also observed a later attention-dependent gamma response localized to the amygdala, presumably due to feedback from prefrontal regions (Luo et al., 2010). However, these studies, as with any EEG or MEG study reporting activation from deep, subcortical structures, should be considered respect to the current limitations in source analysis techniques. Although greater for supraliminally-presented facial expressions, gamma synchronization is also observed in response to facial expressions processed subliminally (Balconi and Lucchiari, 2008; Luo et al., 2009), suggesting that gamma synchronization can be influenced by emotion even in the absence of visual awareness.

These findings are supported by intracranial studies showing localized gamma synchronization in brain regions implicated in emotion processing—most notably, the amygdala and OFC. Recording intracranial field potentials from the amygdala of pre-surgical epileptic patients, Sato et al. (2011) found increased gamma synchronization in the amygdala for fearful compared to neutral facial expressions. The early time course of gamma synchronization (50–150 ms) supports the presence of a subcortical pathway involved in the rapid detection of emotionally salient facial features. Gamma synchronization has also been observed over prefrontal cortices during the later stage of emotional face perception. Consistent with findings from functional neuroimaging studies demonstrating functional subdivisions between medial and lateral regions of the OFC (i.e., Kringelbach and Rolls, 2004), gamma responses in the lateral OFC are greater in response to negative emotions (Jung et al., 2011). However, this effect only occurred when attention was explicitly directed to the emotional quality of the expressions. Thus, during an implicit processing task, no responses in the lateral OFC were observed. Moreover, Jung et al. (2011) observed increased gamma band activity in the medial OFC only in response to target stimuli, regardless of emotional valence. These finding suggest that the medial-lateral distinction between sub-regions of the OFC cannot be explained simply in terms of valence but may instead reflect the processing of relative value within the context of the current environment. Recent studies have also observed differential effects of attention on gamma band activity in a network of brain regions the amygdala and OFC during the perception of emotional facial expressions (Müsch et al., 2014). Thus, gamma synchronization in the OFC may reflect the attention-dependent binding of emotionally salient stimuli with internal representations of their motivational significance.

### Summary

Taken together, the current evidence supports the idea that the perception of emotional facial expressions is mediated by the synchronization of neural oscillations across multiple frequency bands (Güntekin and Başar, 2014). Overall, it appears that lower frequency bands may coordinate patterns of long-range connectivity necessary for the encoding and selection of emotionally salient facial features while higher frequency bands may be associated with the integration of these features at multiple stages of emotion processing.

## Perception of Emotion from Vocal Expressions

Within the auditory domain, emotion can be communicated via affective bursts (laughs, screams, cries, etc.) or more subtle changes in tone of voice, or emotion prosody. While both convey important affective information, perception of emotion from these two types of vocal expressions occurs along different time scales and may rely on different patterns of neural activity and connectivity. Although very few studies have investigated the role of neural oscillations in perception of emotion from either type of vocal expression, current evidence suggests that theta synchronization may play a particularly important role in facilitating the detection of emotionally salient vocal cues.

### Detection of Prosodic Change

A considerable body of research suggests that theta band oscillations drive the processing of slow acoustic changes in speech perception (Peelle and Davis, 2012). To explore the role of oscillatory activity in the detection of emotional prosodic change, Chen et al. (2012) used a cross-splicing procedure to artificially combine vocalizations spoken in angry and neutral prosodies. Thus, vocalizations could change from neutral to angry, angry to neutral, or remain constant. In this paradigm, detection of prosodic change was associated with an increase in fronto-central theta synchronization between 100–600 ms. Furthermore, for angry prosodies only, theta synchronization was modulated by intensity with greater power for high compared to low intensity vocalizations. Subsequent research by the same group has extended these findings, showing increased theta synchronization for neutral to angry change compared to no change for both implicit and explicit tasks suggesting that the emotional content of the stimulus may facilitate the detection of acoustic change (Chen et al., 2014). In this study, significant beta desynchronization was also observed between 400–750 ms, but only when the task required explicit processing of emotional change, which the authors interpret as re-integration of the cross-spliced portion of the sentence with its preceding context. Although these findings provide preliminary support for the role of theta synchronization and beta desynchronization in the detection of emotion prosody, the precise temporal and spatial dynamics of these effects needs to be addressed in order to provide a better characterization of the function of these frequency bands in vocal emotion perception.

### Oscillatory Response to Affective Bursts

With regards to affective bursts, what little evidence there is suggests that gender differences may also influence theta band activity. In a study by Bekkedal et al. (2011), the authors found no main effect of emotion on frontal theta synchronization. Instead, they found an interaction between emotion and gender such that women showed increased theta synchronization for angry expressions over bilateral anterior regions while men showed increased theta synchronization for expressions of pleasure over right anterior regions. As noted by the authors, this gender difference in theta synchronization may be due to differences in arousal, although behavioral measures would certainly be needed to support this claim. Moreover, the wide time intervals used for analysis (500 ms) make the functional interpretation of these gender differences in theta synchronization difficult and may partially account for the absence of any statistically significant differences in other frequency bands.

### Summary

Though few in number, the existing studies suggest that theta synchronization may facilitate the perception of emotion from vocal expressions. Consistent with findings from the speech literature, theta synchronization appears to mediate the detection of acoustic change, an effect which is modulated by emotion. Additionally, beta desynchronization may also play a role in vocal emotion perception, but only when explicitly attending to the change in prosody. Thus, theta synchronization may be involved in the detection of emotionally significant acoustic features during vocal emotion perception while beta desynchronization may facilitate the integration of these features with contextual information.

## Integration of Facial, Body, and Vocal Expressions of Emotion

In natural environments, emotion perception requires the integration of emotional cues from both visual and auditory modalities. Based on current models of visual and auditory emotion perception, it could be hypothesized that multisensory emotional expressions are integrated in a convergent manner such that visual and auditory cues are processed separately in modality-specific cortices, integrated into a coherent multisensory percept the STS, and evaluated in the PFC. However, it is important to note that facial and vocal expressions occur along different temporal scales with changes in facial expression often preceding changes in vocal expressions. Therefore, based on dynamic changes in facial and body expressions, the brain can generate predictions about the timing and content of forthcoming vocal expressions. Evidence from ERP studies suggests that emotional facial expressions elicit stronger (i.e., more reliable) predictions than neutral expressions (Jessen and Kotz, 2011; Jessen et al., 2012; Ho et al., 2015; Kokinous et al., 2015), resulting in facilitated processing of predicted emotional vocalizations. Together with recent proposals suggesting that neural oscillations play an important role in multisensory processing (Schroeder et al., 2008; Senkowski et al., 2008; Arnal and Giraud, 2012), this suggests that neural synchronization may facilitate the processing of multisensory emotional expressions through: (i) the selective binding of emotionally-salient sensory input from different modalities; and (ii) the formation and modification of sensory predictions.

### Multisensory Integration of Facial and Vocal Expressions

Many earlier studies of multisensory emotion perception relied on the use of static facial expressions paired with words or phrases spoken in emotional or neutral prosody. In one such study, Chen et al. (2010) sought to determine whether multisensory integration effects could be observed in the primary sensory cortices during emotional face-voice processing. Using MEG, the authors recorded changes in oscillatory activity during visual, auditory, and audiovisual processing of emotional expressions. However, no integration effects were observed in either visual or auditory cortices. While this finding is interpreted as absence of audiovisual integration in primary sensory cortices, it could also be explained by the absence of predictive visual information since visual and auditory cues were presented simultaneously (see Vroomen and Stekelenburg, 2010). Interestingly, however, the authors observed alpha synchronization over superior frontal and cingulate cortices, which may suggest that increasing the amount of information available to the sensory systems via multiple modalities reduces the cognitive demand on prefrontal regions (Schelenz et al., 2013). Other studies using static facial expressions have found cross-modal interactions in other frequency bands and brain regions. For instance, by presenting participants with static fearful and neutral facial expressions paired with congruent vocal expressions, Hagan et al. (2009) demonstrated supra-additive increases in oscillatory activity in the STS, with theta and gamma bands contributing most to the increase in broadband activity. Subsequent research by the same group showed that supra-additive increases in the STS occurred in both congruent and incongruent conditions (albeit later in the incongruent condition), suggesting automatic integration of emotional facial and vocal expressions (Hagan et al., 2013). Consistent with these findings, other studies have observed theta synchronization during the integration of facial and prosodic change (Chen et al., 2015). Together, these findings suggest that oscillatory activity in the alpha and theta frequency bands drive the integration of facial and vocal expressions. Thus, without predictive visual information, theta synchronization in the STS may facilitate the feedforward integration of visual and auditory input into a coherent percept, reducing the processing demands on prefrontal regions involved in the interpretation and evaluation of the expression.

### Cross-Modal Predictive Coding of Emotional Expressions

Although these studies using static facial expressions have undoubtedly contributed to our understanding of audiovisual integration of emotional expression, their findings could be challenged on the grounds of ecological validity. Therefore, more recent studies have moved towards the use of dynamic facial, body, and vocal expression in order to explore the oscillatory correlates of emotion perception in more naturalistic environments. In among the first to do so, Jessen and Kotz (2011) presented participants with video clips of dynamic facial, body, and vocal expressions. Using EEG, the authors found significant decreases in both alpha and beta power for audiovisual compared to the sum of auditory- and visual-only conditions with additional suppression for emotional compared to neutral expressions. These findings were replicated in a subsequent study, which also showed that while beta suppression for the contrast between multimodal and unimodal conditions was localized to the premotor cortex, suppression for the contrast between emotional and neutral conditions was localized to the posterior parietal cortex (Jessen et al., 2012). Since previous studies have demonstrated beta suppression in these regions during the processing of biological motion (Muthukumaraswamy et al., 2006; Muthukumaraswamy and Singh, 2008), it could be argued that the observed differences in beta power are due to differences in the motion content between emotional and neutral expressions. However, for the stimuli used in these studies, there was no difference in the motion content before the onset of the vocal expression (see Jessen and Kotz, 2011) making it unlikely that beta suppression was an artifact of differences in motion content. Instead, beta oscillations may play a broader role in the predictive coding of audiovisual information (i.e., Arnal and Giraud, 2012). Furthermore, the observed differences in beta ERD between emotional and neutral expressions provide support for the hypothesis that emotional expressions generate stronger cross-modal predictions compared to neutral expressions (Jessen and Kotz, 2013).

### Summary

Taken together, these studies support previous research suggesting that neural oscillations play an important role in multisensory processing. Furthermore, these findings show that the emotional content of the stimulus may facilitate flexible integration of facial, body, and vocal expressions. The simultaneous presentation of visual and auditory expressions results in synchronization of theta oscillations in the STS (i.e., the STS) and alpha oscillations over prefrontal regions, suggesting that theta synchronization mediates the integration of audiovisual emotional expressions. Previous evidence suggests that multimodal expressions generally are more easily recognizable than unimodal expressions (Collignon et al., 2008; Tanaka et al., 2010; Föcker et al., 2011), frontal alpha synchronization may reflect relative inhibition of regions needed to resolve any remaining uncertainty with regards to the emotional content of the stimulus. Since this effect was observed in emotion categorization tasks, it is possible that different task demands will induce different spatial and temporal patterns of alpha synchronization. In contrast, the natural temporal delay between visual and auditory expressions enables the brain use changes in facial and body expression to generate predictions about the timing and content of forthcoming vocal expressions. Thus, cross-modal prediction results in ERD, particularly in the alpha and beta frequencies. These findings support the idea that multisensory integration and cross-modal prediction are distinct yet interactive mechanisms underpinning the multisensory emotion perception (Jessen and Kotz, 2013, 2015).

## Discussion

Nonverbal emotion perception is driven by dynamic, context-dependent interactions within and between brain regions involved in the detection, integration, and evaluation of emotional expressions. Where and when such interactions occur depends on the sensory modality (or modalities) through which the emotion is expressed as well as the emotional quality of the stimulus itself. However, emotional expressions are dynamic events that continuously evolve over time. Therefore, the neural system(s) supporting emotion perception must be able to flexibly adapt to and integrate rapidly changing sensory input from multiple modalities. Based on the reviewed evidence, we propose that neural synchronization underpins the selective attention to and the flexible binding of emotionally salient sensory input across different spatial and temporal scales. Furthermore, neural oscillations provide a mechanism through which emotional facial and body expressions can predictively modulate the processing of subsequent vocal expressions.

The recognition of an expression as “emotional” requires the selective binding of emotionally relevant sensory information. However, individual features of an emotional expression can occur at different points in time and are processed in spatially distinct regions of the brain. Thus, the brain is challenged with the task of binding only those features belonging to the same event across both space and time. One mechanism through which this may occur is the synchronization of neural oscillations, which creates temporal windows in which information belonging to the same event can be selected and integrated (Singer and Gray, 1995). Moreover, coherence between distinct neuronal populations may enable the flexible neuronal communication across different regions of the brain (Fries, 2005). Consistent with this idea, current evidence suggests that the synchronization of neural oscillations supports the selection and integration of sensory information within and between modalities (Senkowski et al., 2008; van Atteveldt et al., 2014). Gamma band oscillations, in particular, are thought to be important for sensory binding and feature integration on a local scale (Tallon-Baudry and Bertrand, 1999). As previously discussed, perception of emotion from facial expressions results in increased gamma band synchronization, suggesting that gamma band oscillations may mediate the rapid integration of emotionally salient sensory input. However, gamma band synchronization may be modulated by lower-frequency oscillations. Since lower frequency bands represent the activity of larger neuronal populations and longer temporal windows, such cross-frequency coupling between low and high frequency oscillations may enable the integration of information across different spatial and temporal scales (Canolty and Knight, 2010).

Natural communicative signals exhibit strong regularities that enable the brain to generate predictions about forthcoming sensory information within and between sensory modalities. This process may be mediated by the functional coupling of neural oscillations, which can facilitate the efficient allocation of processing resources to the predicted sensory input. For instance, synchronization of low-frequency oscillations may coordinate the allocation of processing resources, via high-frequency oscillations, at the phase in which the predicted sensory input occurs (Hyafil et al., 2015). As an example, the natural temporal delay between visual and acoustic speech signals provides a means through which the visual signal can alter the phase of ongoing neural oscillations such that the expected acoustic signal occurs at the phase of optimal neuronal excitability (Schroeder et al., 2008). While the phase of low-frequency oscillations may create temporal windows for the selection of relevant sensory information, higher-frequency beta and gamma oscillations may be involved in the transmission of top-down predictions (both formal and temporal) and bottom-up prediction errors, respectively (Arnal et al., 2011; Arnal and Giraud, 2012). If this is indeed the case, then it follows that neural oscillations, particularly within these frequency bands, may facilitate the predictive coding of nonverbal communicative signals such as dynamic facial, body, and vocal expressions. In this respect, emotion perception is similar to other forms of perception, with emotion acting as a highly salient source of relevant information that must be encoded and integrated with other sources of sensory information.

## Future Directions

### Effect of Modality

Although early on, Charles Darwin recognized the equal importance of facial, body, and vocal expressions in emotional communication, research over the past 50 years has focused predominantly on the perception of emotion from facial expressions. Thus, the role of neural oscillations in emotion perception has primarily been studied by presenting participants with images of static facial expressions. While this approach has yielded some valuable results, it does not necessarily reflect how emotions are expressed and perceived in natural human communication.

In everyday life, emotional expressions are dynamic, characterized by changes in facial expression, body language, and prosody unfolding over time. To this end, previous functional neuroimaging research has shown distinct neural pathways involved in the perception of emotion from static and dynamic facial expressions (i.e., Kilts et al., 2003). Consistent with these findings, results from Jabbi et al. (2015) suggest that oscillatory activity in the beta frequency band may track dynamic changes in sensory input facilitating the differentiation of emotional expressions. Although the use of dynamic facial expressions adds an additional level of stimulus complexity, it also affords greater ecological validity, which can improve our understanding of the neural dynamics underpinning naturalistic emotion perception. Moreover, the dynamic nature of emotional expressions enables the brain to use incoming sensory input to generate predictions about future events. Future studies using methods such as dynamic causal modeling (DCM) can be used to compare convergent and predictive coding models of multisensory emotion perception.

A second issue relates to the fact that facial expressions are also not the only means of emotional communication. Changes in emotional body language (De Gelder, 2006) and prosody (Schirmer and Kotz, 2006) also provide important information about one’s emotional state. Compared to facial expressions, however, little is known about the oscillatory dynamics underpinning the perception of emotion from body and vocal expressions. Therefore, further research into: (i) the perception of emotion from dynamic body and vocal expressions; and (ii) the integration of emotional expressions from multiple modalities is needed if we are to understand the neural bases of emotion perception in human social interactions.

### Emotional Differentiation

Each emotion is associated with a unique physiological, cognitive, and behavioral profile that serves an adaptive and, in social species, a communicative function. Therefore, it is likely that distinct patterns of neural activity and connectivity drive the expression and perception of different emotions.

One of the broadest distinctions between emotions is that of valence, which categorizes emotions as positive (pleasant) or negative (unpleasant). Within the brain, some have proposed that the right hemisphere is dominant for the processing of negative emotions while the left hemisphere is dominant for positive emotions (Ahern and Schwartz, 1979, 1985; Silberman and Weingartner, 1986). Although valence-specific asymmetry has primarily been discussed within the context of emotional experience, studies in healthy individuals and in patients with unilateral brain damage suggest that there may also be hemispheric asymmetry in the perception of emotion (i.e., Jansari et al., 2000; Adolphs et al., 2001), though this may be influenced by task demands (Kotz et al., 2003, 2006). Consistent with this hypothesis, there is preliminary support for valence-specific hemispheric asymmetry of alpha desynchronization during the emotion perception (i.e., Balconi and Ferrari, 2012). However, given support for alternative hypotheses such as the approach-withdrawal model of hemispheric lateralization (Davidson, 1992), future studies examining patterns of coherence across brain regions during the perception of positive and negative emotions are needed in order to elucidate the functional dynamics underpinning the differentiation of emotional valence.

Since each emotion serves a distinct function, it has been hypothesized that there may be different, yet partially overlapping, neural pathways specialized for the processing of different emotions (i.e., LeDoux, 2000). Thus, we may expect specific patterns of neural synchronization during the perception of different emotions. In support of this idea, distinct spatial and temporal patterns of theta (Knyazev et al., 2009b) and gamma (Luo et al., 2007) band activity have been observed in response to different emotions. So although perception of different emotions may rely partially overlapping networks, further investigations into patterns of neural synchronization and coherence may reveal subtle changes in functional dynamics that enable us to differentiate between emotions.

### Individual Differences

Due to the interaction between neurophysiological and environmental factors, individual differences can have a profound effect on how we perceive and interpret nonverbal expressions of emotion. Underlying these individual differences are changes in functional coupling that can be investigated by examining patterns of neural synchrony. To this end, gender differences are reflected in beta (Güntekin and Başar, 2007b) and theta (Knyazev et al., 2010) synchronization in response to emotional facial expressions. Furthermore, alpha desynchronization has been negatively associated with extraversion (Fink, 2005) and hostility (Knyazev et al., 2009b) and positively associated with anxiety (Knyazev et al., 2008) and depression (Knyazev et al., 2015). Individual differences have also been observed in the theta band, with reduced frontal theta synchronization in individuals with high levels of anxiety (Knyazev et al., 2008) and depression (Knyazev et al., 2015) and increased theta synchronization in those scoring high on measures of emotional intelligence (Knyazev et al., 2013). Additionally, hostility has been associated with gender differences in alpha and theta synchronization over posterior regions (Knyazev et al., 2009b) while dominance motivation is associated with delta/beta asymmetry (Hofman et al., 2013). Taken together, these findings suggest that changes in patterns of neural synchronization may mediate individual differences in the perception of emotional expressions.

### Clinical Implications

Deficits in the ability to accurately perceive and interpret emotions have been observed in a number of neurological and psychiatric conditions, the neural bases of which remain poorly understood. By enabling us to look beyond the activity of specific brain regions into the dynamics of functional neural networks, investigations into changes in neural synchronization and coherence can advance our understanding of the specific impairments associated with different clinical conditions. Work in this area has already begun with studies showing reduced theta synchronization during perception of emotional facial expressions in individuals with schizophrenia (Ramos-Loyo et al., 2009; Csukly et al., 2014). Schizophrenia has also been associated with abnormal patterns of alpha synchronization (Ramos-Loyo et al., 2009; Popov et al., 2014), though this may be improved through targeted training in facial affect recognition (Popova et al., 2014). Other studies have found that oscillatory responses to facial expressions in the gamma band differentiate between unipolar and bipolar depression; while individuals with unipolar depression show reduced gamma power in response to sad facial expressions, those with bipolar show increased gamma band activity in response to highly arousing emotions (Liu et al., 2014). Finally, adolescents with Autism Spectrum Disorder show reduced interregional beta synchronization in response to angry facial expressions, suggesting that impairments in functional connectivity within networks involved in emotion processing may contribute to the deficits in facial emotion perception observed in autism (Leung et al., 2014). Thus, a better characterization of oscillatory responses to emotional expressions may aid in the diagnosis and treatment of a number of clinical conditions.

## Conclusion

From the reviewed studies, it is clear that the perception of facial, body, and vocal expressions of emotion is mediated by oscillatory activity in multiple frequency bands. Although research on delta synchronization has been primarily restricted to the visual domain, the important delta oscillations and their functional coupling with higher (beta/gamma) frequency bands, in basic biological, cognitive, and emotional processes highlights the need for further research into the functional role of delta oscillations in emotion perception within and between sensory modalities. Across modalities, theta synchronization most consistently differentiates between emotional and neutral expressions and may reflect the initial encoding and derivation of emotional significance. Changes in alpha power have been primarily observed in studies with a visual component, with some evidence of valence-specific lateralization over frontal regions. Based on the hypothesis that alpha synchronization reflects active inhibition of task irrelevant brain regions (Klimesch et al., 2007), modulation of alpha power may reflect sensory selection and inhibition of behaviorally relevant sensory information. Although evidence is still inconclusive as to the role of beta oscillations in emotion perception, changes in beta power are more likely to be observed in studies using dynamic stimuli or in those involving shifts in attention, consistent with the idea that beta band activity reflects the maintenance of the current cognitive or sensorimotor set (Engel and Fries, 2010). Gamma synchronization has been observed in emotion processing regions such as the amygdala, STS, and OFC, suggesting that oscillatory activity in this frequency band is associated with the binding of emotionally salient sensory input. The modulation of specific frequency bands by emotion enables the selective detection, integration, and evaluation of emotional signals through coordinated changes in effective connectivity. From a predictive coding perspective, the emotional quality of the expression may act as a particularly salient source of information, strengthening the precision of sensory predictions through enhanced neural synchronization. However, further research, particularly in the auditory and audiovisual domains, is clearly necessary to gain a deeper understanding of the neural dynamics underpinning the perception of emotion within and between sensory modalities.

## Author Contributions

Main contribution by first author (AES). All the other authors contributed equally to this work.

## Conflict of Interest Statement

The authors declare that the research was conducted in the absence of any commercial or financial relationships that could be construed as a potential conflict of interest.

## References

[B1] Adolphs R. (2002a). Neural systems for recognizing emotion. Curr. Opin. Neurobiol. 12, 169–177. 10.1016/s0959-4388(02)00301-x 12015233

[B2] Adolphs R. (2002b). Recognizing emotion from facial expressions: psychological and neurological mechanisms. Behav. Cogn. Neurosci. Rev. 1, 21–62. 10.1177/1534582302001001003 17715585

[B3] Adolphs R. Jansari A. Tranel D. (2001). Hemispheric perception of emotional valence from facial expressions. Neuropsychology 15, 516–524. 10.1037/0894-4105.15.4.516 11761041

[B4] Ahern G. L. Schwartz G. E. (1979). Differential lateralization for positive versus negative emotion. Neuropsychologia 17, 693–698. 10.1016/0028-3932(79)90045-9 522984

[B5] Ahern G. L. Schwartz G. E. (1985). Differential lateralization for positive and negative emotion in the human brain: EEG spectral analysis. Neuropsychologia 23, 745–755. 10.1016/0028-3932(85)90081-8 4080136

[B6] Arnal L. H. Giraud A. L. (2012). Cortical oscillations and sensory predictions. Trends Cogn. Sci. 16, 390–398. 10.1016/j.tics.2012.05.003 22682813

[B8] Arnal L. H. Wyart V. Giraud A. L. (2011). Transitions in neural oscillations reflect prediction errors generated in audiovisual speech. Nat. Neurosci. 14, 797–801. 10.1038/nn.2810 21552273

[B9] Balconi M. Ferrari C. (2012). Subliminal and supraliminal processing of facial expression of emotions: brain oscillation in the left/right frontal area. Brain Sci. 2, 85–100. 10.3390/brainsci2020085 24962767 PMC4061794

[B10] Balconi M. Lucchiari C. (2006). EEG correlates (event-related desynchronization) of emotional face elaboration: a temporal analysis. Neurosci. Lett. 392, 118–123. 10.1016/j.neulet.2005.09.004 16202519

[B11] Balconi M. Lucchiari C. (2008). Consciousness and arousal effects on emotional face processing as revealed by brain oscillations. A γ band analysis. Int. J. Psychophysiol. 67, 41–46. 10.1016/j.ijpsycho.2007.10.002 17997495

[B12] Balconi M. Pozzoli U. (2007). Event-related oscillations (EROs) and event-related potentials (ERPs) comparison in facial expression recognition. J. Neuropsychol. 1, 283–294. 10.1348/174866407x184789 19331021

[B13] Balconi M. Pozzoli U. (2008). Event-related oscillations (ERO) and event-related potentials (ERP) in emotional face recognition. Int. J. Neurosci. 118, 1412–1424. 10.1080/00207450601047119 18788026

[B14] Balconi M. Pozzoli U. (2009). Arousal effect on emotional face comprehension. Frequency band changes in different time intervals. Physiol. Behav. 97, 455–462. 10.1016/j.physbeh.2009.03.023 19341748

[B15] Başar E. Güntekin B. Öniz A. (2006). Principles of oscillatory brain dynamics and a treatise of recognition of faces and facial expressions. Prog. Brain Res. 159, 43–62. 10.1016/s0079-6123(06)59004-1 17071223

[B16] Bastiaansen M. Mazaheri A. Jensen O. (2012). “Beyond ERPs: oscillatory neuronal dynamics,” in The Oxford Handbook of Event-Related Potential Components, eds Luck S. J. Kappenman E. S. (New York, NY: Oxford University Press), 31–50.

[B17] Beauchamp M. S. Argall B. D. Bodurka J. Duyn J. H. Martin A. (2004a). Unraveling multisensory integration: patchy organization within human STS multisensory cortex. Nat. Neurosci. 7, 1190–1192. 10.1038/nn1333 15475952

[B18] Beauchamp M. S. Lee K. E. Argall B. D. Martin A. (2004b). Integration of auditory and visual information about objects in superior temporal sulcus. Neuron 41, 809–823. 10.1016/s0896-6273(04)00070-4 15003179

[B19] Bekkedal M. Y. V. Rossi J. III Panksepp J. (2011). Human brain EEG indices of emotions: delineating responses to affective vocalizations by measuring frontal theta event-related synchronization. Neurosci. Biobehav. Rev. 35, 1959–1970. 10.1016/j.neubiorev.2011.05.001 21596060

[B20] Belin P. Fecteau S. Bédard C. (2004). Thinking the voice: neural correlates of voice perception. Trends Cogn. Sci. 8, 129–135. 10.1016/j.tics.2004.01.008 15301753

[B21] Berger H. (1929). Über das elektrenkephalogramm des menschen. Eur. Arch. Psychiatry Clin. Neurosci. 87, 527–570. 10.1007/bf01797193

[B22] Blair R. J. R. Morris J. S. Frith C. D. Perrett D. I. Dolan R. J. (1999). Dissociable neural responses to facial expressions of sadness and anger. Brain 122, 883–893. 10.1093/brain/122.5.883 10355673

[B23] Brovelli A. Ding M. Ledberg A. Chen Y. Nakamura R. Bressler S. L. (2004). α oscillations in a large-scale sensorimotor cortical network: directional influences revealed by Granger causality. Proc. Natl. Acad. Sci. U S A 101, 9849–9854. 10.1073/pnas.0308538101 15210971 PMC470781

[B24] Brück C. Kreifelts B. Wildgruber D. (2011). Emotional voices in context: a neurobiological model of multimodal affective information processing. Phys. Life Rev. 8, 383–403. 10.1016/j.plrev.2011.10.002 22035772

[B25] Calvert G. A. Campbell R. Brammer M. J. (2000). Evidence from functional magnetic resonance imaging of crossmodal binding in the human heteromodal cortex. Curr. Biol. 10, 649–657. 10.1016/s0960-9822(00)00513-3 10837246

[B26] Calvert G. A. Hansen P. C. Iversen S. D. Brammer M. J. (2001). Detection of audio-visual integration sites in humans by application of electrophysiological criteria to the BOLD effect. Neuroimage 14, 427–438. 10.1006/nimg.2001.0812 11467916

[B27] Canolty R. T. Knight R. T. (2010). The functional role of cross-frequency coupling. Trends Cogn. Sci. 14, 506–515. 10.1016/j.tics.2010.09.001 20932795 PMC3359652

[B31] Chen Y. H. Edgar J. C. Holroyd T. Dammers J. Thönneßen H. Roberts T. P. L.. (2010). Neuromagnetic oscillations to emotional faces and prosody. Eur. J. Neurosci. 31, 1818–1827. 10.1111/j.1460-9568.2010.07203.x 20584186

[B28] Chen X. Pan Z. Wang P. Yang X. Liu P. You X.. (2015). The integration of facial and vocal cues during emotional change perception: EEG markers. Soc. Cogn. Affect. Neurosci. [Epub ahead of print]. 10.1093/scan/nsv083 26130820 PMC4927038

[B29] Chen X. Pan Z. Wang P. Zhang L. Yuan J. (2014). EEG oscillations reflect task effects for the change detection in vocal emotion. Cogn. Neurodyn. 9, 351–358. 10.1007/s11571-014-9326-9 25972983 PMC4427591

[B30] Chen X. Yang J. Gan S. Yang Y. (2012). The contribution of sound intensity in vocal emotion perception: behavioral and electrophysiological evidence. PLoS One 7:e30278. 10.1371/journal.pone.0030278 22291928 PMC3264585

[B32] Collignon O. Girard S. Gosselin F. Roy S. Saint-Amour D. Lassonde M.. (2008). Audio-visual integration of emotion expression. Brain Res. 1242, 126–135. 10.1016/j.brainres.2008.04.023 18495094

[B33] Csukly G. Stefanics G. Komlósi S. Czigler I. Czobor P. (2014). Event-related theta synchronization predicts deficit in facial affect recognition in schizophrenia. J. Abnorm. Psychol. 123, 178–189. 10.1037/a0035793 24661169

[B34] Davidson R. J. (1992). Anterior cerebral asymmetry and the nature of emotion. Brain Cogn. 20, 125–151. 10.1016/0278-2626(92)90065-t 1389117

[B35] De Gelder B. (2006). Towards the neurobiology of emotional body language. Nat. Rev. Neurosci. 7, 242–249. 10.1038/nrn1872 16495945

[B36] Del Zotto M. Deiber M. P. Legrand L. B. De Gelder B. Pegna A. J. (2013). Emotional expressions modulate low alpha and α oscillations in a cortically blind patient. Int. J. Psychophysiol. 90, 358–362. 10.1016/j.ijpsycho.2013.10.007 24144636

[B37] Eimer M. Holmes A. (2002). An ERP study on the time course of emotional face processing. Neuroreport 13, 427–431. 10.1097/00001756-200203250-00013 11930154

[B38] Eimer M. Holmes A. (2007). Event-related brain potential correlates of emotional face processing. Neuropsychologia 45, 15–31. 10.1016/j.neuropsychologia.2006.04.022 16797614 PMC2383989

[B39] Eimer M. Holmes A. McGlone F. P. (2003). The role of spatial attention in the processing of facial expression: an ERP study of rapid brain responses to six basic emotions. Cogn. Affect. Behav. Neurosci. 3, 97–110. 10.3758/cabn.3.2.97 12943325

[B40] Engel A. K. Fries P. (2010). β-band oscillations-signalling the status quo? Curr. Opin. Neurobiol. 20, 156–165. 10.1016/j.conb.2010.02.015 20359884

[B41] Ethofer T. Bretscher J. Gschwind M. Kreifelts B. Wildgruber D. Vuilleumier P. (2011). Emotional voice areas: anatomic location, functional properties and structural connections revealed by combined fMRI/DTI. Cereb. Cortex 22, 191–200. 10.1093/cercor/bhr113 21625012

[B42] Ethofer T. Van De Ville D. Scherer K. Vuilleumier P. (2009). Decoding of emotional information in voice-sensitive cortices. Curr. Biol. 19, 1028–1033. 10.1016/j.cub.2009.04.054 19446457

[B43] Fecteau S. Belin P. Joanette Y. Armony J. L. (2007). Amygdala responses to nonlinguistic emotional vocalizations. Neuroimage 36, 480–487. 10.1016/j.neuroimage.2007.02.043 17442593

[B44] Fink A. (2005). Event-related desynchronization in the EEG during emotional and cognitive information processing: differential effects of extraversion. Biol. Psychol. 70, 152–160. 10.1016/j.biopsycho.2005.01.013 16198045

[B45] Föcker J. Gondan M. Röder B. (2011). Preattentive processing of audio-visual emotional signals. Acta Psychol. (Amst) 137, 36–47. 10.1016/j.actpsy.2011.02.004 21397889

[B46] Fries P. (2005). A mechanism for cognitive dynamics: neuronal communication through neuronal coherence. Trends Cogn. Sci. 9, 474–480. 10.1016/j.tics.2005.08.011 16150631

[B47] Fries P. Schröder J.-H. Roelfsema P. R. Singer W. Engel A. K. (2002). Oscillatory neuronal synchronization in primary visual cortex as a correlate of stimulus selection. J. Neurosci. 22, 3739–3754. 11978850 10.1523/JNEUROSCI.22-09-03739.2002 PMC6758402

[B48] Frühholz S. Grandjean D. (2013a). Processing of emotional vocalizations in bilateral inferior frontal cortex. Neurosci. Biobehav. Rev. 37, 2847–2855. 10.1016/j.neubiorev.2013.10.007 24161466

[B49] Frühholz S. Grandjean D. (2013b). Amygdala subregions differentially respond and rapidly adapt to threatening voices. Cortex 49, 1394–1403. 10.1016/j.cortex.2012.08.003 22938844

[B50] Fulford D. Peckham A. D. Johnson K. Johnson S. L. (2014). Emotion perception and quality of life in bipolar I disorder. J. Affect. Disord. 152–154, 491–497. 10.1016/j.jad.2013.08.034 24070906 PMC3851889

[B51] Garrido-Vásquez P. Jessen S. Kotz S. A. (2011). Perception of emotion in psychiatric disorders: on the possible role of task, dynamics and multimodality. Soc. Neurosci. 6, 515–536. 10.1080/17470919.2011.620771 21961831

[B52] González-Roldan A. M. Martínez-Jauand M. Muñoz-García M. A. Sitges C. Cifre I. Montoya P. (2011). Temporal dissociation in the brain processing of pain and anger faces with different intensities of emotional expression. Pain 152, 853–859. 10.1016/j.pain.2010.12.037 21296497

[B53] Grèzes J. Pichon S. de Gelder B. (2007). Perceiving fear in dynamic body expressions. Neuroimage 35, 959–967. 10.1016/j.neuroimage.2006.11.030 17270466

[B54] Güntekin B. Başar E. (2007a). Emotional face expressions are differentiated with brain oscillations. Int. J. Psychophysiol. 64, 91–100. 10.1016/j.ijpsycho.2006.07.003 17156875

[B55] Güntekin B. Başar E. (2007b). Gender differences influence brain’s α oscillatory responses in recognition of facial expressions. Neurosci. Lett. 424, 94–99. 10.1016/j.neulet.2007.07.052 17716816

[B56] Güntekin B. Başar E. (2009). Facial affect manifested by multiple oscillations. Int. J. Psychophysiol. 71, 31–36. 10.1016/j.ijpsycho.2008.07.019 18725252

[B57] Güntekin B. Başar E. (2014). A review of brain oscillations in perception of faces and emotional pictures. Neuropsychologia 58, 33–51. 10.1016/j.neuropsychologia.2014.03.014 24709570

[B58] Güntekin B. Başar E. (2015). Review of evoked and event-related delta responses in the human brain. Int. J. Psychophysiol. [Epub ahead of print]. 10.1016/j.ijpsycho.2015.02.001 25660301

[B59] Hadjikhani N. de Gelder B. (2003). Seeing fearful body expressions activates the fusiform cortex and amygdala. Curr. Biol. 13, 2201–2205. 10.1016/j.cub.2003.11.049 14680638

[B60] Hagan C. C. Woods W. Johnson S. Calder A. J. Green G. G. R. Young A. W. (2009). MEG demonstrates a supra-additive response to facial and vocal emotion in the right superior temporal sulcus. Proc. Natl. Acad. Sci. U S A 106, 20010–20015. 10.1073/pnas.0905792106 19906999 PMC2785283

[B61] Hagan C. C. Woods W. Johnson S. Green G. G. R. Young A. W. (2013). Involvement of right STS in audio-visual integration for affective speech demonstrated using MEG. PLoS One 8:e70648. 10.1371/journal.pone.0070648 23950977 PMC3741276

[B62] Ho H. T. Schröger E. Kotz S. A. (2015). Selective attention modulates early human evoked potentials during emotional face-voice processing. J. Cogn. Neurosci. 27, 798–818. 10.1162/joc_a_00734 25269113

[B63] Hofman D. Terburg D. van Wielink L. Schutter D. J. L. G. (2013). Coalescence of dominance motivation and responses to facial anger in resting-state and event-related electrophysiology. Neuroimage 79, 138–144. 10.1016/j.neuroimage.2013.04.088 23644002

[B64] Hyafil A. Giraud A. L. Fontolan L. Gutkin B. (2015). Neural cross-frequency coupling: connecting architectures, mechanisms and functions. Trends Neurosci. 38, 725–740. 10.1016/j.tins.2015.09.001 26549886

[B65] Jabbi M. Kohn P. D. Nash T. Ianni A. Coutlee C. Holroyd T.. (2015). Convergent BOLD and α-band activity in superior temporal sulcus and frontolimbic circuitry underpins human emotion cognition. Cereb. Cortex 25, 1878–1888. 10.1093/cercor/bht427 24464944 PMC4459288

[B66] Jansari A. Tranel D. Adolphs R. (2000). A valence-specific lateral bias for discriminating emotional facial expressions in free field. Cogn. Emot. 14, 341–353. 10.1080/026999300378860

[B67] Jensen O. Mazaheri A. (2010). Shaping functional architecture by oscillatory alpha activity: gating by inhibition. Front. Hum. Neurosci. 4:186. 10.3389/fnhum.2010.00186 21119777 PMC2990626

[B68] Jessen S. Kotz S. A. (2011). The temporal dynamics of processing emotions from vocal, facial and bodily expressions. Neuroimage 58, 665–674. 10.1016/j.neuroimage.2011.06.035 21718792

[B69] Jessen S. Kotz S. A. (2013). On the role of crossmodal prediction in audiovisual emotion perception. Front. Hum. Neurosci. 7:369. 10.3389/fnhum.2013.00369 23882204 PMC3714569

[B70] Jessen S. Kotz S. A. (2015). Affect differentially modulates brain activation in uni-and multisensory body-voice perception. Neuropsychologia 66, 134–143. 10.1016/j.neuropsychologia.2014.10.038 25445782

[B71] Jessen S. Obleser J. Kotz S. A. (2012). How bodies and voices interact in early emotion perception. PLoS One 7:e36070. 10.1371/journal.pone.0036070 22558332 PMC3340409

[B72] Jung J. Bayle D. Jerbi K. Vidal J. R. Hénaff M. A. Ossandon T.. (2011). Intracerebral γ modulations reveal interaction between emotional processing and action outcome evaluation in the human orbitofrontal cortex. Int. J. Psychophysiol. 79, 64–72. 10.1016/j.ijpsycho.2010.09.014 20933545

[B73] Kilts C. D. Egan G. Gideon D. A. Ely T. D. Hoffman J. M. (2003). Dissociable neural pathways are involved in the recognition of emotion in static and dynamic facial expressions. Neuroimage 18, 156–168. 10.1006/nimg.2002.1323 12507452

[B74] Klimesch W. (1999). EEG alpha and theta oscillations reflect cognitive and memory performance: a review and analysis. Brain Res. Rev. Res. Rev. 29, 169–195. 10.1016/s0165-0173(98)00056-3 10209231

[B75] Klimesch W. Sauseng P. Hanslmayr S. (2007). EEG alpha oscillations: the inhibition-timing hypothesis. Brain Res. Rev. 53, 63–88. 10.1016/j.brainresrev.2006.06.003 16887192

[B76] Knyazev G. G. (2007). Motivation, emotion and their inhibitory control mirrored in brain oscillations. Neurosci. Biobehav. Rev. 31, 377–395. 10.1016/j.neubiorev.2006.10.004 17145079

[B77] Knyazev G. G. (2012). EEG delta oscillations as a correlate of basic homeostatic and motivational processes. Neurosci. Biobehav. Rev. 36, 677–695. 10.1016/j.neubiorev.2011.10.002 22020231

[B79] Knyazev G. G. Bocharov A. V. Levin E. A. Savostyanov A. N. Slobodskoj-Plusnin J. Y. (2008). Anxiety and oscillatory responses to emotional facial expressions. Brain Res. 1227, 174–188. 10.1016/j.brainres.2008.06.108 18639533

[B80] Knyazev G. G. Bocharov A. V. Savostyanov A. N. Slobodskoy-Plusnin J. (2015). Predisposition to depression and implicit emotion processing. J. Clin. Exp. Neuropsychol. 37, 701–709. 10.1080/13803395.2015.1061483 26207798

[B82] Knyazev G. G. Bocharov A. V. Slobodskoj-Plusnin J. Y. (2009a). Hostility- and gender-related differences in oscillatory responses to emotional facial expressions. Aggress. Behav. 35, 502–513. 10.1002/ab.20318 19670288

[B81] Knyazev G. G. Mitrofanova L. G. Bocharov A. V. (2013). Emotional intelligence and oscillatory responses to emotional facial expressions. Hum. Physiol. 39, 371–377. 10.1134/s0362119713030110 25486829

[B78] Knyazev G. G. Slobodskoj-Plusnin J. Y. Bocharov A. V. (2009b). Event-related delta and theta synchronization during explicit and implicit emotion processing. Neuroscience 164, 1588–1600. 10.1016/j.neuroscience.2009.09.057 19796666

[B83] Knyazev G. G. Slobodskoj-Plusnin J. Y. Bocharov A. V. (2010). Gender differences in implicit and explicit processing of emotional facial expressions as revealed by event-related theta synchronization. Emotion 10, 678–687. 10.1037/a0019175 21038950

[B84] Kokinous J. Kotz S. A. Tavano A. Schröger E. (2015). The role of emotion in dynamic audiovisual integration of faces and voices. Soc. Cogn. Affect. Neurosci. 10, 713–720. 10.1093/scan/nsu105 25147273 PMC4420746

[B85] Kotz S. A. Hasting A. S. Paulmann S. (2012). “On the orbito-striatal interface in (acoustic) emotional processing,” in The Evolution of Emotional Communication: From Sounds in Nonhuman Mammals to Speech and Music in Man, eds Altenmüller E. Schmidt S. Zimmermann E. (New York, NY: Oxford University Press), 229–240.

[B87] Kotz S. A. Meyer M. Alter K. Besson M. von Cramon D. Y. Friederici A. D. (2003). On the lateralization of emotional prosody: an event-related functional MR investigation. Brain Lang. 86, 366–376. 10.1016/s0093-934x(02)00532-1 12972367

[B86] Kotz S. A. Meyer M. Paulmann S. (2006). Lateralization of emotional prosody in the brain: an overview and synopsis on the impact of study design. Prog. Brain Res. 156, 285–294. 10.1016/s0079-6123(06)56015-7 17015086

[B88] Kotz S. A. Paulmann S. (2011). Emotion, language and the brain. Lang. Linguist. Compass 5, 108–125. 10.1111/j.1749-818x.2010.00267.x

[B89] Kreifelts B. Ethofer T. Shiozawa T. Grodd W. Wildgruber D. (2009). Cerebral representation of non-verbal emotional perception: fMRI reveals audiovisual integration area between voice- and face-sensitive regions in the superior temporal sulcus. Neuropsychologia 47, 3059–3066. 10.1016/j.neuropsychologia.2009.07.001 19596021

[B90] Kret M. E. Pichon S. Grèzes J. de Gelder B. (2011). Similarities and differences in perceiving threat from dynamic faces and bodies. An fMRI study. Neuroimage 54, 1755–1762. 10.1016/j.neuroimage.2010.08.012 20723605

[B91] Kringelbach M. L. Rolls E. T. (2004). The functional neuroanatomy of the human orbitofrontal cortex: evidence from neuroimaging and neuropsychology. Prog. Neurobiol. 72, 341–372. 10.1016/j.pneurobio.2004.03.006 15157726

[B92] LeDoux J. (1996). The Emotional Brain: The Mysterious Underpinnings of Emotional Life. New York, NY: Simon and Schuster.

[B93] LeDoux J. E. (2000). Emotion circuits in the brain. Annu. Rev. Neurosci. 23, 155–184. 10.1146/annurev.neuro.23.1.155 10845062

[B94] Leung R. C. Ye A. X. Wong S. M. Taylor M. J. Doesburg S. M. (2014). Reduced α connectivity during emotional face processing in adolescents with autism. Mol. Autism 5:51. 10.1186/2040-2392-5-51 25371811 PMC4218990

[B95] Liu T. Y. Chen Y. S. Su T. P. Hsieh J. C. Chen L. F. (2014). Abnormal early γ responses to emotional faces differentiate unipolar from bipolar disorder patients. Biomed Res. Int. 2014:906104. 10.1155/2014/906104 24738077 PMC3971502

[B96] Liu T. Pinheiro A. P. Deng G. Nestor P. G. McCarley R. W. Niznikiewicz M. A. (2012). Electrophysiological insights into processing nonverbal emotional vocalizations. Neuroreport 23, 108–112. 10.1097/WNR.0b013e32834ea757 22134115

[B97] Luo Q. Holroyd T. Jones M. Hendler T. Blair J. (2007). Neural dynamics for facial threat processing as revealed by γ band synchronization using MEG. Neuroimage 34, 839–847. 10.1016/j.neuroimage.2006.09.023 17095252 PMC1839041

[B98] Luo Q. Holroyd T. Majestic C. Cheng X. Schechter J. Blair R. J. (2010). Emotional automaticity is a matter of timing. J. Neurosci. 30, 5825–5829. 10.1523/JNEUROSCI.BC-5668-09.2010 20427643 PMC3842481

[B99] Luo Q. Mitchell D. Cheng X. Mondillo K. Mccaffrey D. Holroyd T.. (2009). Visual awareness, emotion and γ band synchronization. Cereb. Cortex 19, 1896–1904. 10.1093/cercor/bhn216 19047574 PMC2705698

[B100] Meeren H. K. de Gelder B. Ahlfors S. P. Hämäläinen M. S. Hadjikhani N. (2013). Different cortical dynamics in face and body perception: an MEG study. PLoS One 8:e71408. 10.1371/journal.pone.0071408 24039712 PMC3765413

[B101] Müsch K. Hamamé C. M. Perrone-Bertolotti M. Minotti L. Kahane P. Engel A. K. (2014). Selective attention modulates high-frequency activity in the face-processing network. Cortex 60, 34–51. 10.1016/j.cortex.2014.06.006 25017647

[B103] Muthukumaraswamy S. D. Johnson B. W. Gaetz W. C. Cheyne D. O. (2006). Neural processing of observed oro-facial movements reflects multiple action encoding strategies in the human brain. Brain Res. 1071, 105–112. 10.1016/j.brainres.2005.11.053 16405872

[B102] Muthukumaraswamy S. D. Singh K. D. (2008). Modulation of the human mirror neuron system during cognitive activity. Psychophysiology 45, 896–905. 10.1111/j.1469-8986.2008.00711.x 18823416

[B105] Paulmann S. Bleichner M. Kotz S. A. (2013). Valence, arousal and task effects in emotional prosody processing. Front. Psychol. 4:345. 10.3389/fpsyg.2013.00345 23801973 PMC3689289

[B104] Paulmann S. Kotz S. A. (2008). Early emotional prosody perception based on different speaker voices. Neuroreport 19, 209–213. 10.1097/WNR.0b013e3282f454db 18185110

[B106] Paulmann S. Jessen S. Kotz S. A. (2012). It’s special the way you say it: an ERP investigation on the temporal dynamics of two types of prosody. Neuropsychologia 50, 1609–1620. 10.1016/j.neuropsychologia.2012.03.014 22465251

[B107] Pavlova M. A. (2012). Biological motion processing as a hallmark of social cognition. Cereb. Cortex 22, 981–995. 10.1093/cercor/bhr156 21775676

[B108] Peelle J. E. Davis M. H. (2012). Neural oscillations carry speech rhythm through to comprehension. Front. Psychol. 3:320. 10.3389/fpsyg.2012.00320 22973251 PMC3434440

[B109] Pell M. D. Rothermich K. Liu P. Paulmann S. Sethi S. Rigoulot S. (2015). Preferential decoding of emotion from human non-linguistic vocalizations versus speech prosody. Biol. Psychol. 111, 14–25. 10.1016/j.biopsycho.2015.08.008 26307467

[B110] Pernet C. R. McAleer P. Latinus M. Gorgolewski K. J. Charest I. Bestelmeyer P. E. G.. (2015). The human voice areas: spatial organisation and inter-individual variability in temporal and extra-temporal cortices. Neuroimage 119, 164–174. 10.1016/j.neuroimage.2015.06.050 26116964 PMC4768083

[B111] Péron J. Biseul I. Leray E. Vicente S. Le Jeune F. Drapier S.. (2010a). Subthalamic nucleus stimulation affects fear and sadness recognition in Parkinson’s disease. Neuropsychology 24, 1-8. 10.1037/a0017433 20063943

[B112] Péron J. Frühholz S. Vérin M. Grandjean D. (2013). Subthalamic nucleus: a key structure for emotional component synchronization in humans. Neurosci. Biobehav. Rev. 37, 358–373. 10.1016/j.neubiorev.2013.01.001 23318227

[B113] Péron J. Grandjean D. Le Jeune F. Sauleau P. Haegelen C. Drapier D.. (2010b). Recognition of emotional prosody is altered after subthalamic nucleus deep brain stimulation in Parkinson’s disease. Neuropsychologia 48, 1053–1062. 10.1016/j.neuropsychologia.2009.12.003 20005239

[B114] Pfurtscheller G. Stancák A. Neuper C. (1996). Event-related synchronization (ERS) in the alpha band - an electrophysiological correlate of cortical idling: a review. Int. J. Psychophysiol. 24, 39–46. 10.1016/s0167-8760(96)00066-9 8978434

[B117] Phillips M. L. Drevets W. C. Rauch S. L. Lane R. (2003). Neurobiology of emotion perception II: implications for major psychiatric disorders. Biol. Psychiatry 54, 515–528. 10.1016/s0006-3223(03)00171-9 12946880

[B115] Phillips L. H. Henry J. D. Scott C. Summers F. Whyte M. Cook M. (2011). Specific impairments of emotion perception in multiple sclerosis. Neuropsychology 25, 131–136. 10.1037/a0020752 21090898

[B116] Phillips L. H. Scott C. Henry J. D. Mowat D. Bell J. S. (2010). Emotion perception in Alzheimer’s disease and mood disorder in old age. Psychol. Aging 25, 38–47. 10.1037/a0017369 20230126

[B118] Phillips M. L. Young A. W. Senior C. Brammer M. Andrew C. Calder A. J.. (1997). A specific neural substrate for perceiving facial expressions of disgust. Nature 389, 495–498. 10.1038/39051 9333238

[B120] Popov T. Miller G. A. Rockstroh B. Weisz N. (2013). Modulation of α power and functional connectivity during facial affect recognition. J. Neurosci. 33, 6018–6026. 10.1523/JNEUROSCI.2763-12.2013 23554483 PMC6618940

[B121] Popova P. Popov T. G. Wienbruch C. Carolus A. M. Miller G. A. Rockstroh B. S. (2014). Changing facial affect recognition in schizophrenia: effects of training on brain dynamics. Neuroimage Clin. 6, 156–165. 10.1016/j.nicl.2014.08.026 25379427 PMC4215531

[B119] Popov T. G. Rockstroh B. S. Popova P. Carolus A. M. Miller G. A. (2014). Dynamics of alpha oscillations elucidate facial affect recognition in schizophrenia. Cogn. Affect. Behav. Neurosci. 14, 364–377. 10.3758/s13415-013-0194-2 23943514

[B122] Ramos-Loyo J. González-Garrido A. A. Sánchez-Loyo L. M. Medina V. Basar-Eroglu C. (2009). Event-related potentials and event-related oscillations during identity and facial emotional processing in schizophrenia. Int. J. Psychophysiol. 71, 84–90. 10.1016/j.ijpsycho.2008.07.008 18727940

[B123] Robins D. L. Hunyadi E. Schultz R. T. (2009). Superior temporal activation in response to dynamic audio-visual emotional cues. Brain Cogn. 69, 269–278. 10.1016/j.bandc.2008.08.007 18809234 PMC2677198

[B124] Rolls E. T. (2004). The functions of the orbitofrontal cortex. Brain Cogn. 55, 11–29. 10.1016/S0278-2626(03)00277-X 15134840

[B125] Sato W. Kochiyama T. Uono S. Matsuda K. Usui K. Inoue Y.. (2011). Rapid amygdala γ oscillations in response to fearful facial expressions. Neuropsychologia 49, 612–617. 10.1016/j.neuropsychologia.2010.12.025 21182851

[B126] Schelenz P. D. Klasen M. Reese B. Regenbogen C. Wolf D. Kato Y.. (2013). Multisensory integration of dynamic emotional faces and voices: method for simultaneous EEG-fMRI measurements. Front. Hum. Neurosci. 7:729. 10.3389/fnhum.2013.00729 24294195 PMC3827626

[B127] Scherer K. R. (2000). “Psychological models of emotion” in The Neuropsychology of Emotion, ed J. Board (Oxford, NY: Oxford University Press), 137–162.

[B128] Schirmer A. Kotz S. A. (2006). Beyond the right hemisphere: brain mechanisms mediating vocal emotional processing. Trends Cogn. Sci. 10, 24–30. 10.1016/j.tics.2005.11.009 16321562

[B129] Schirmer A. Striano T. Friederici A. D. (2005). Sex differences in the preattentive processing of vocal emotional expressions. Neuroreport 16, 635–639. 10.1097/00001756-200504250-00024 15812323

[B131] Schroeder C. E. Lakatos P. Kajikawa Y. Partan S. Puce A. (2008). Neuronal oscillations and visual amplification of speech. Trends Cogn. Sci. 12, 106–113. 10.1016/j.tics.2008.01.002 18280772 PMC3987824

[B132] Schutter D. J. L. G. Putman P. Hermans E. van Honk J. (2001). Parietal electroencephalogram α asymmetry and selective attention to angry facial expressions in healthy human subjects. Neurosci. Lett. 314, 13–16. 10.1016/s0304-3940(01)02246-7 11698135

[B133] Schwarzlose R. F. Baker C. I. Kanwisher N. (2005). Separate face and body selectivity on the fusiform gyrus. J. Neurosci. 25, 11055–11059. 10.1523/jneurosci.2621-05.2005 16306418 PMC6725864

[B134] Senkowski D. Schneider T. R. Foxe J. J. Engel A. K. (2008). Crossmodal binding through neural coherence: implications for multisensory processing. Trends Neurosci. 31, 401–409. 10.1016/j.tins.2008.05.002 18602171

[B135] Silberman E. K. Weingartner H. (1986). Hemispheric lateralization of functions related to emotion. Brain Cogn. 5, 322–353. 10.1016/0278-2626(86)90035-7 3530287

[B136] Singer W. Gray C. M. (1995). Visual feature integration and the temporal correlation hypothesis. Annu. Rev. Neurosci. 18, 555–586. 10.1146/annurev.neuro.18.1.555 7605074

[B137] Stefanics G. Csukly G. Komlósi S. Czobor P. Czigler I. (2012). Processing of unattended facial emotions: a visual mismatch negativity study. Neuroimage 59, 3042–3049. 10.1016/j.neuroimage.2011.10.041 22037000

[B139] Stevenson R. A. Geoghegan M. L. James T. W. (2007). Superadditive BOLD activation in superior temporal sulcus with threshold non-speech objects. Exp. Brain Res. 179, 85–95. 10.1007/s00221-006-0770-6 17109108

[B138] Stevenson R. A. James T. W. (2009). Audiovisual integration in human superior temporal sulcus: inverse effectiveness and the neural processing of speech and object recognition. Neuroimage 44, 1210–1223. 10.1016/j.neuroimage.2008.09.034 18973818

[B140] Tallon-Baudry C. Bertrand O. (1999). Oscillatory γ activity and its role in object representation. Trends Cogn. Sci. 3, 151–162. 10.1016/s1364-6613(99)01299-1 10322469

[B141] Tanaka A. Koizumi A. Imai H. Hiramatsu S. Hiramoto E. de Gelder B. (2010). I feel your voice. Cultural differences in the multisensory perception of emotion. Psychol. Sci. 21, 1259–1262. 10.1177/0956797610380698 20713633

[B142] van Atteveldt N. Murray M. M. Thut G. Schroeder C. E. (2014). Multisensory integration: flexible use of general operations. Neuron 81, 1240–1253. 10.1016/j.neuron.2014.02.044 24656248 PMC4090761

[B143] Vroomen J. Stekelenburg J. J. (2010). Visual anticipatory information modulates multisensory interactions of artificial audiovisual stimuli. J. Cogn. Neurosci. 22, 1583–1596. 10.1162/jocn.2009.21308 19583474

[B144] Vuilleumier P. Pourtois G. (2007). Distributed and interactive brain mechanisms during emotion face perception: evidence from functional neuroimaging. Neuropsychologia 45, 174–194. 10.1016/j.neuropsychologia.2006.06.003 16854439

[B145] Watson R. Latinus M. Noguchi T. Garrod O. Crabbe F. Belin P. (2013). Dissociating task difficulty from incongruence in face-voice emotion integration. Front. Hum. Neurosci. 7:744. 10.3389/fnhum.2013.00744 24294196 PMC3826561

[B146] Watson R. Latinus M. Noguchi T. Garrod O. Crabbe F. Belin P. (2014). Crossmodal adaptation in right posterior superior temporal sulcus during face-voice emotional integration. J. Neurosci. 34, 6813–6821. 10.1523/JNEUROSCI.4478-13.2014 24828635 PMC4019796

[B147] Whalen P. J. Raila H. Bennett R. Mattek A. Brown A. Taylor J. (2013). Neuroscience and facial expressions of emotion: the role of amygdala-prefrontal interactions. Emot. Rev. 5, 78–83. 10.1177/1754073912457231

[B148] Whalen P. J. Shin L. M. McInerney S. C. Fischer H. Wright C. I. Rauch S. L. (2001). A functional MRI study of human amygdala responses to facial expressions of fear versus anger. Emotion 1, 70-83. 10.1037/1528-3542.1.1.70 12894812

[B149] Wiethoff S. Wildgruber D. Kreifelts B. Becker H. Herbert C. Grodd W.. (2008). Cerebral processing of emotional prosody—influence of acoustic parameters and arousal. Neuroimage 39, 885–893. 10.1016/j.neuroimage.2007.09.028 17964813

[B150] Wildgruber D. Ethofer T. Grandjean D. Kreifelts B. (2009). A cerebral network model of speech prosody comprehension. Int. J. Speech Lang. Pathol. 11, 277–281. 10.1080/17549500902943043

[B151] Williams M. A. Morris A. P. McGlone F. Abbott D. F. Mattingley J. B. (2004). Amygdala responses to fearful and happy facial expressions under conditions of binocular suppression. J. Neurosci. 24, 2898–2904. 10.1523/jneurosci.4977-03.2004 15044528 PMC6729857

[B152] Woods D. L. Alain C. (2009). Functional imaging of human auditory cortex. Curr. Opin. Otolaryngol. Head Neck Surg. 17, 407–411. 10.1097/MOO.0b013e3283303330 19633556

[B153] Zhang D. Wang L. Luo Y. Luo Y. (2012). Individual differences in detecting rapidly presented fearful faces. PLoS One 7:e49517. 10.1371/journal.pone.0049517 23166693 PMC3498139

